# Andrographolide Suppresses Proliferation of Nasopharyngeal Carcinoma Cells via Attenuating NF-**κ**B Pathway

**DOI:** 10.1155/2015/735056

**Published:** 2015-03-15

**Authors:** Tao Peng, Min Hu, Ting-Ting Wu, Cen Zhang, Zhe Chen, Shuo Huang, Xu-Hong Zhou

**Affiliations:** Department of Otorhinolaryngology, Head and Neck Surgery, Zhongnan Hospital of Wuhan University, 169 Donghu Road, Wuhan, Hubei 430071, China

## Abstract

Andrographolide (Andro) has been reported to have anticancer activity in multiple types of cancer due to its capacity to inactivate NF-*κ*B pathway. Previous studies showed the therapeutic potential of targeting NF-*κ*B pathway in nasopharyngeal carcinoma (NPC). However, the anticancer activity of Andro in NPC has not been reported. In this study, we defined the anticancer effects of Andro in NPC and elucidated its potential mechanisms of action. Our results showed that Andro significantly inhibited the proliferation and invasion of NPC cells (*P* < 0.05, resp.). These anticancer activities were associated with cell apoptosis, cell death and induction of cell cycle arrest, and the downregulation of NF-*κ*B target genes. This work provides evidence that NF-*κ*B pathway is a potential therapeutic target and may also be indispensable in the Andro-mediated anticancer activities in nasopharyngeal carcinoma.

## 1. Introduction

Nasopharyngeal carcinoma (NPC) is a southern China-prevalent head and neck cancer, with an annual incidence of 15–50 cases per 100,000 [1]. NPC has been characterized by poorly or undifferentiated carcinoma, and recurrence and distant metastasis remain the major causes for NPC deaths [2]. Despite initial responses, the benefits of conventional therapies are seldom long-term and the toxicities are intolerable for most patients [3]. Therefore, specific targeted therapies for NPC have become the focus of development. Recent studies indicated that NF-*κ*B is commonly activated in NPC [4, 5], and treatments with NF-*κ*B inhibitors such as dehydroxymethylepoxyquinomicin (DHMEQ) may inhibit the growth and invasion of NPC [2]. Currently, it is believed that NF-*κ*B is a potential molecular target for NPC therapy. Andrographolide (Andro) is a major bioactive component isolated from* Andrographis paniculata* [6], which has been used to treat infections and inflammatory diseases [7]. Recent studies reported that Andro possesses anticancer activities in several types of cancer due to its capacity to inactivate NF-*κ*B pathways [8, 9]. Thus, we hypothesized that Andro may exhibit anticancer effects in NPC via inactivating NF-*κ*B. However, there was no study especially investigating the anticancer effects of Andro in NPC. In the present study, we aim to investigate whether Andro has anticancer effects on NPC and the mechanism by which Andro inhibits growth and invasion of NPC cells.

## 2. Materials and Methods

### 2.1. Ethics Statement

This study was conducted in the Center for Medical Experiment of Zhongnan Hospital of Wuhan University (Wuhan, Hubei, China) from March to November 2014. The Research Ethics Committee of Zhongnan Hospital of Wuhan University has approved this study. All procedures were performed in accordance with the National Research Council Guide. The Research Ethics Committee of Zhongnan Hospital approved our protocol numbered 2014ZN009 in March 2014.

### 2.2. Cell Culture and Regents

#### 2.2.1. Cell Culture

As previously described [10], all NPC cells were cultured in RPMI1640 medium (Sigma, St. Louis, MO, USA), supplemented with 10% FBS (MP Biomedicals, OH), 5% glutamine, 100 U/mL penicillin, and 100 mg/mL streptomycin (Invitrogen, Carlsbad, CA). All NPC cell lines were purchased from The Medical Experiment Center of Zhongnan Hospital of Wuhan University (Wuhan, Hubei, China). Cultures were maintained at 37°C in a humidified atmosphere of 95% air and 5% CO_2_.

Andro was purchased from Aldrich (Milwaukee, WI, USA); Bay 11-7082 was purchased from Calbiochem (NJ, USA). Dimethyl sulfoxide (DMSO) was from Sigma-Aldrich (St. Louis, MO, USA). TNF-alpha was purchased from R&D Co. (Minneapolis, MN, USA). Antibodies including anti-survivin, anti-cyclin D1, anti-ICAM-1, anti-EGFR, anti-MMP-9, and anti-VEGF were purchased from Abcam (Shanghai, China). The secondary antibodies for Western blot were purchased from Boster (Wuhan, China).

### 2.3. NF-*κ*B Luciferase Assay

As previously described [2], NF-*κ*B luciferase plasmid (NF-*κ*B-Luc) was constructed with DNA sequencing confirmation. Transient transfected NPC cells (HK1 and CNE-1) were lysed with luciferase lysis buffer. Luciferase activity was measured as previously described [11].

### 2.4. Cell Viability Assay

Cell viability was determined using the tetrazolium dye 3-(4,5-dimethylthiazol-2-yl)-2,5-diphenyltetrazolium bromide (MTT) assay as previously described [12]. Briefly, cells plated in the 96-well plates were treated with Andro at indicated concentrations (5, 10, and 25 *μ*M). Cells treated with DMSO were used as the control group. At 24, 48, and 72 h after treatments, cell viability was determined.

### 2.5. Cell Death and Apoptosis Analysis

NPC cells (56104) were seeded in each well of the culture plate and treated with Andro at indicated concentrations (5 and 25 *μ*M) for 24 h. Flow cytometry was used to determine cell apoptosis. Annexin V-positive and PI-negative cells were identified as apoptotic cells. Cells with or without Andro treatment were washed once with PBS and stained with phycoerythrin (PE)/Annexin V before flow analysis. The apoptotic rate was determined using CellQuest software (FCM, BD Biosciences, San Jose, CA, USA). Cell death was determined using a* Cell Death Detection ELISA* ^*PLUS*^ kit (Roche Life Science, China) according to manufacturer's instructions after a 24 h treatment with Andro or DMSO.

### 2.6. Cell Cycle Analysis

NPC cells were treated with Andro (25 *μ*M) or DMSO (final concentration in cell culture was 1%) in complete medium for 14 h. Then, cells were fixed with 70% ethanol and stained with propidium iodide (PI) (0.05 *μ*g/mL, Sigma, St. Louis, MO, USA) solution containing RNase A (0.2 mg/mL). Analysis was performed using a FACScan flow cytometer (Becton Dickinson, Bedford, MA).

### 2.7. Colony Formation Assay

NPC cells were treated with DMSO (final concentration in cell culture was 1%) or Andro at indicated concentrations (5, 10, and 25 *μ*M). Cells were incubated at 37°C for 2 weeks, and then cells were washed twice with PBS and stained with crystal violet staining solution. The number of colonies was counted under microscope. The colony formation efficiency was calculated as follows: colony formation efficiency = (number of colonies/number of cells inoculated) × 100%.

### 2.8. Cell Invasion Assay

Cell invasion was assayed in a cell culture chamber (BD, Bedford, MA) with 8 *μ*m pore size polycarbonate membrane filters. Cells were seeded onto the upper chamber and maintained in serum-free medium. The cell-containing chamber was immersed in lower chamber containing medium with 10% FBS, with or without Andro treatment. Cells were incubated at 37°C for 24 h. Invaded cells were stained and counted as previously described [13].

### 2.9. Western Blot Analysis

Cells were treated with Andro, DMSO, or Bay 11-7082 at indicated doses for 24 h, and the cell lysates were subjected to Western blots using the antibodies of survivin, ICAM-1, cyclin D1, EGFR, MMP-9, and VEGF. The protein bands were normalized with *β*-Action, and all blots were quantified with Quantity One software (Bio-Rad).

### 2.10. Statistical Analysis

Data were analyzed using SPSS software version 21.0 (SPSS Inc., Chicago, IL). Comparisons among all groups were performed with the one-way analysis of variance (ANOVA) test or unpaired Student's *t*-test. A *P* value less than 0.5 was considered significant.

## 3. Results

### 3.1. Andro Inhibited NF-*κ*B Transcriptional Activity in NPC Cells

**Figure 1:**
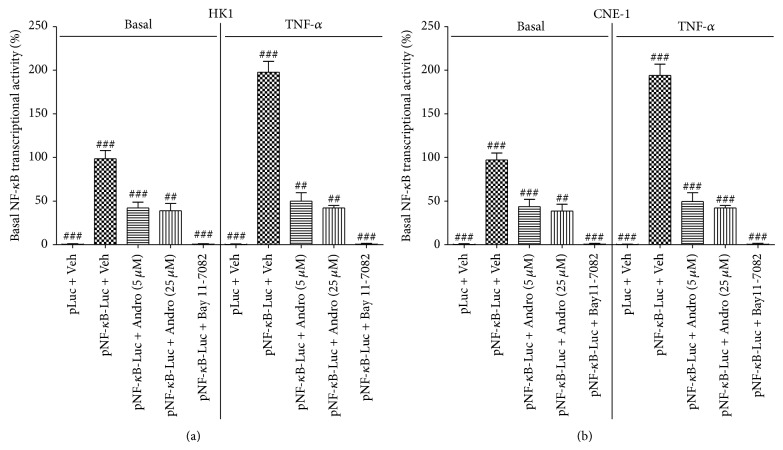
Andro inhibited the transcriptional activity of NF-*κ*B in HK1 (a) and CNE-1 (b) cells. Cells were pretreated with Andro at different concentrations (5 and 25 *μ*M) for 3 h and further incubated with or without TNF-*α* for additional 6 h. Bay 11-7082, a NF-*κ*B inhibitor, was included as a positive control. Compared with the respective control, ^##^ *P* < 0.01 and ^###^ *P* < 0.001.

In this study, we used a luciferase reporter assay to examine the effects of Andro on NF-*κ*B transcriptional activity in NPC cell lines, HK1 and CNE-1. Bay 11-7082, an inhibitor of NF-*κ*B, has been used as a positive control. As shown in Figure 1, control vector transfection showed a lowest luciferase activity, while transfection of pNF-*κ*B-Luc into NPC cells resulted in significantly higher NF-*κ*B transcriptional activities for both HK1 (*P* < 0.001) and CNE-1 (*P* < 0.001) cell lines. Treatment with TNF-*α* (30 ng/mL) enhanced NF-*κ*B-dependent transcription in the pNF-*κ*B-Luc transfectants (*P* < 0.0001) but not in the control (*P* = 0.27). When pNF-*κ*B-Luc transfectants were treated with Andro (5 *μ*M or 25 *μ*M), both basal NF-*κ*B-dependent transcriptional activity and TNF-*α*-induced NF-*κ*B transcriptional activity have been significantly reduced in NPC cell lines including HK1 (*P* = 0.0054 and 0.0029, resp.) and CNE-1 (*P* = 0.00018 and 0.0024, resp.).

### 3.2. Andro Inhibited Proliferation and Invasion of NPC Cells

**Figure 2:**
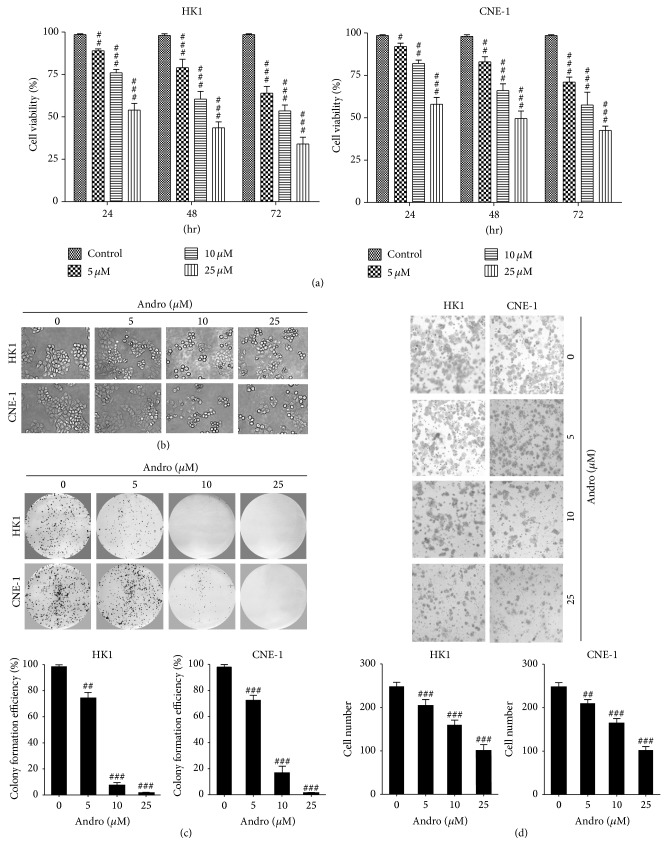
Andro inhibited the proliferation and invasion of different NPC cell lines. (a) Andro inhibited NPC cell proliferation. (b) NPC cells were treated with Andro at indicated doses for 24 h. Cell morphology was observed by the phase contrast microscopy. (c) The colony formation efficiency was detected by the plate clone formation assay. (d) Andro inhibited cell invasion in different NPC cell lines. The average numbers of migrated cells per field were presented as mean ± SD (*n* = 10 fields). Compared with the control (DMSO), ^#^ *P* < 0.05, ^##^ *P* < 0.01, and ^###^ *P* < 0.001.

To further evaluate the antiproliferative effects of Andro on NPC cells, the cells were cultured with Andro at indicated concentrations (5, 10, and 25 *μ*M) for 24, 48, and 72 h, respectively. As shown in Figure 2(a), Andro was capable of inhibiting the growth of HK1 and CNE-1 cells in a time- and dose-dependent manner. When cells were intubated with 25 *μ*M Andro for 72 h, the growth inhibition achieved 72.8% in HK1 cells and 70.5% in CNE-1 cells. Additionally, the treatments with Andro caused the distinct cellular morphological changes including shrinking, rounding, and detachment from the adjacent cells (Figure 2(b)). Moreover, Andro significantly inhibited the colony formation efficiency of HK1 (*P* < 0.001, resp.) and CNE-1 (*P* < 0.0001, resp.) cells (Figure 2(c)). Next, we investigated the effects of Andro on cell invasiveness by cell invasion assay (Figure 2(d)). Andro (25 *μ*M) reduced the transwell invasion of HK1 and CNE-1 cells by 64.2% and 62.5%, respectively.

### 3.3. Andro Induced G2/M Arrest, Cell Death, and Apoptosis in NPC Cells

**Figure 3:**
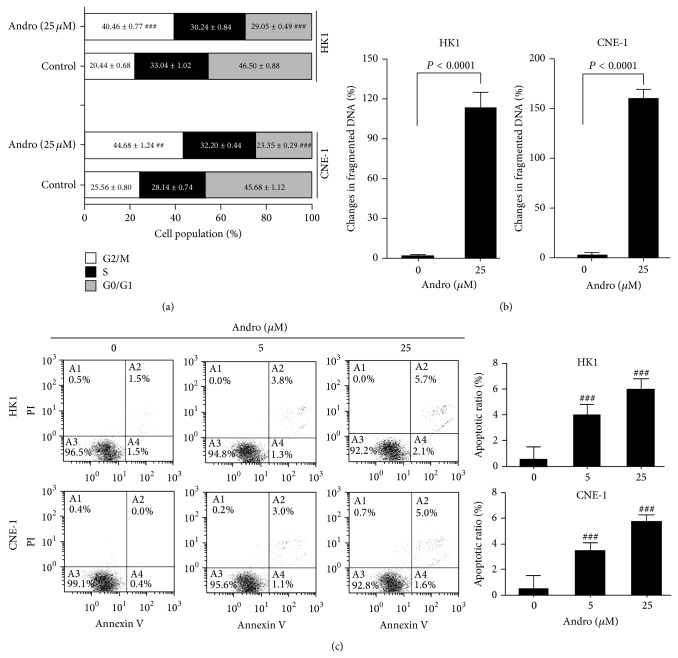
Andro induced G2/M arrest (a), cell death (b), and apoptosis (c) in two different NPC cell lines HK1 and CNE-1. Cells were treated with DMSO or Andro at indicated doses. Percentage of cells was shown as mean ± SD (*n* = 5). Compared with control, ^###^ *P* < 0.001.

As shown in Figure 3(a), Andro induced a significant cell cycle arrest in G2/M phase in both HK1 and CNE-1 cell lines (*P* < 0.001, resp.). In HK1 cells, Andro (25 *μ*M) induced a 20% increase of G2/M phase, accompanied by G0/G1 phase reduction (17%) when compared to control. Similar results were observed in CNE-1 cells. In this study, we detected the fragmentation of DNA (an irreversible event of apoptosis) in NPC cells treated with or without Andro (Figure 3(b)). At 25 *μ*M, Andro treatment resulted in a significant induction of DNA fragmentation of both HK1 and CNE-1 cells (*P* < 0.001, resp.). In addition, the effects of Andro on cell apoptosis were also analyzed (Figure 3(c)). At dose of 5 *μ*M, Andro induced 4.5% and 3.8% apoptosis in HK1 and CNE-1 cells after 24 h treatment, respectively. However, treatment with Andro at 25 *μ*M resulted in the induction of 6.4% and 5.9% apoptosis in HK1 and CNE-1 cells.

### 3.4. Andro Inhibited Expression of NF-*κ*B Target Genes

**Figure 4:**
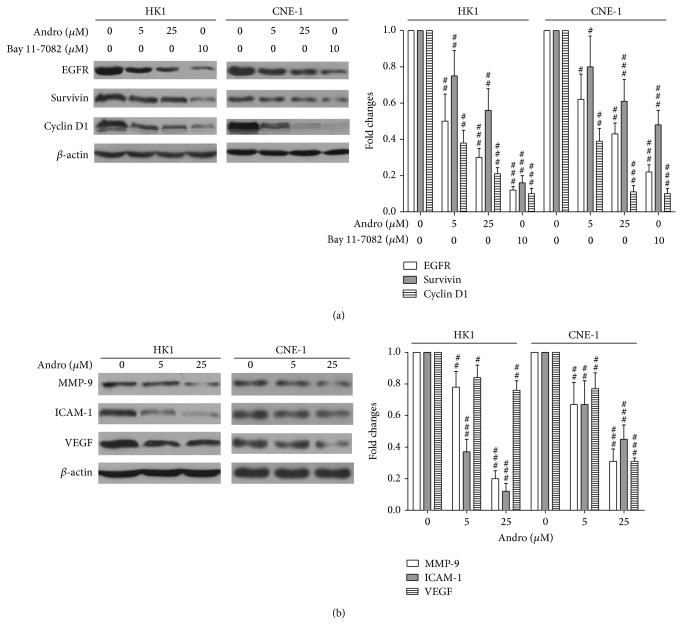
Andro reduced the expression of NF-*κ*B target genes. (a) Andro induced downregulation of NF-*κ*B target genes EGFR, cyclin D1, and survivin. (b) Andro reduced expression of MMP-9, ICAM-1, and VEGF in NPC cell lines. Actin served as the loading control. Compared with control (DMSO) group, ^#^ *P* < 0.05, ^##^ *P* < 0.01, and ^###^ *P* < 0.001.

The expressions of target genes of NF-*κ*B related proliferation and apoptosis were determined in this study. As shown in Figure 4(a), EGFR, survivin, and cyclin D1 were significantly reduced with the treatments of Andro (*P* < 0.001, resp.). In addition, the expressions of all those markers were inhibited by Andro in a dose-dependent manner in both HK1 and CNE-1 cells. Similar inhibitory effects on the expression of those genes were also observed with the positive control Bay 11-7082. Furthermore, we also found downregulation of several invasion or metastasis-related NF-*κ*B target genes including MMP-9, ICAM-1, and VEGF after Andro treatments (Figure 4(b)). Those results indicated that the anti-invasion activity of Andro on NPC cells was possibly associated with downregulation of metastasis-related NF-*κ*B target genes.

## 4. Discussion

In the present study, we demonstrated promising antiproliferation and anti-invasion effects of Andro on NPC cells. The Andro-mediated growth inhibition was observed with the presence of cell cycle arrest at G2/M phase, cell apoptosis induction, and downregulation of the expressions of NF-*κ*B target genes including EGFR, cyclin D1, and survivin. In addition, serum induced cell invasion has been also significantly reduced by Andro. The anti-invasion effects of Andro seemed to be associated with downregulation of MMP-9, VEGF, and ICAM-1. Those findings implicated the importance of targeting NF-*κ*B in NPC cells. However, our* in vitro* findings warrant further* in vivo* evaluation such as metastatic models of NPC.

NF-*κ*B overexpression has been found in several types of cancers and has been indicated as a marker of unfavorable outcomes [14, 15]. Recent studies indicated that NF-*κ*B is also commonly activated in NPC [4, 5], and numerous evidences indicate the involvement of NF-*κ*B in NPC carcinogenesis. Sun et al. reported that overexpression of NF-*κ*B predicts the poor prognosis of NPC [16]. These findings indicated that NF-*κ*B is a potential therapeutic target in NPC. Currently, it is believed that the Epstein-Barr virus (EBV) infection and inflammatory cytokines (such as TNF-*α*) are strong stimuli of NF-*κ*B activation in NPC patients [17, 18]. This study demonstrated the direct functional consequence of targeting NF-*κ*B in NPC. We used Andro or Bay 11-7082 (a NF-*κ*B inhibitor) to inhibit the transcriptional activity of NF-*κ*B. Our results showed that NF-*κ*B activation is required for NPC cell proliferation, invasion, and regulation of cell apoptosis and death. In addition, we also observed that TNF-*α*-induced NF-*κ*B transcriptional activity in NPC cells was blocked by Andro at indicated doses.

Current evidences showed that NF-*κ*B is a major transcriptional factor mediating LMP-1 induced changes of expressions of cancer-related genes including survivin, MMP-9, and EGFR in NPC [19–21]. In addition, NF-*κ*B target genes including survivin, cyclin D1, and EGFR have been known as markers of cell growth and survival, and MMP-9, ICAM-1, and VEGF were considered as metastasis-related NF-*κ*B target genes [2]. In this study, the inhibition of NF-*κ*B transcriptional activity by Andro resulted in decreased expression of NF-*κ*B target genes including survivin, cyclin D1, MMP-9, EGFR, VEGF, and ICAM-1. Thus, the antiproliferation effects of Andro were possibly associated with the downregulation of survivin, cyclin D1, and EGFR in NPC cells, and the anti-invasion effects of Andro may be due to the downregulation of MMP-9, VEGF, and ICAM-1. Those results were consistent with the observation in previous studies on NF-*κ*B inhibition [2, 22].

In this study, Andro induced a significant cell cycle arrest in G2/M phase in this NPC study. Li et al. reported that Andro induced cell cycle arrest at G2/M phase via the alteration of cellular redox status in HepG2 cells [23]. Previous study also reported that Andro inhibits the growth of glioblastoma cells via inducing G2/M arrest, which is mediated by inhibiting the activity of PI3K/Akt signaling [24]. However, the mechanism for the observed Andro-induced G2/M arrest in NPC cell lines remains unclear in this study.

## 5. Conclusion

The results of the present study demonstrated that andrographolide, a novel NF-*κ*B inhibitor, inhibits the proliferation and invasion of NPC cells via suppressing NF-*κ*B transcriptional activity and inhibiting the expression of NF-*κ*B target genes. Thus, andrographolide could be a candidate drug for NPC treatment in the future.
